# Human Leptospirosis Infection in Fiji: An Eco-epidemiological Approach to Identifying Risk Factors and Environmental Drivers for Transmission

**DOI:** 10.1371/journal.pntd.0004405

**Published:** 2016-01-28

**Authors:** Colleen L. Lau, Conall H. Watson, John H. Lowry, Michael C. David, Scott B. Craig, Sarah J. Wynwood, Mike Kama, Eric J. Nilles

**Affiliations:** 1 Children’s Health and Environment Program, Centre for Child Health Research, The University of Queensland, Brisbane, Australia; 2 Queensland Children’s Medical Research Institute, Brisbane, Australia; 3 Research School of Population Health, Australian National University, Canberra, Australia; 4 Centre for the Mathematical Modelling of Infectious Diseases, London School of Hygiene & Tropical Medicine, London, United Kingdom; 5 School of Geography, Earth Science and Environment, University of the South Pacific, Suva, Fiji; 6 WHO/FAO/OIE Collaborating Centre for Reference and Research on Leptospirosis, Forensic and Scientific Services, Health Support Queensland, Department of Health, Brisbane, Australia; 7 Fiji Centre for Communicable Disease Control, Ministry of Health, Suva, Fiji; 8 Division of Pacific Technical Support, World Health Organization, Suva, Fiji; Institut Pasteur, FRANCE

## Abstract

Leptospirosis is an important zoonotic disease in the Pacific Islands. In Fiji, two successive cyclones and severe flooding in 2012 resulted in outbreaks with 576 reported cases and 7% case-fatality. We conducted a cross-sectional seroprevalence study and used an eco-epidemiological approach to characterize risk factors and drivers for human leptospirosis infection in Fiji, and aimed to provide an evidence base for improving the effectiveness of public health mitigation and intervention strategies. Antibodies indicative of previous or recent infection were found in 19.4% of 2152 participants (81 communities on the 3 main islands). Questionnaires and geographic information systems data were used to assess variables related to demographics, individual behaviour, contact with animals, socioeconomics, living conditions, land use, and the natural environment. On multivariable logistic regression analysis, variables associated with the presence of *Leptospira* antibodies included male gender (OR 1.55), iTaukei ethnicity (OR 3.51), living in villages (OR 1.64), lack of treated water at home (OR 1.52), working outdoors (1.64), living in rural areas (OR 1.43), high poverty rate (OR 1.74), living <100m from a major river (OR 1.41), pigs in the community (OR 1.54), high cattle density in the district (OR 1.04 per head/sqkm), and high maximum rainfall in the wettest month (OR 1.003 per mm). Risk factors and drivers for human leptospirosis infection in Fiji are complex and multifactorial, with environmental factors playing crucial roles. With global climate change, severe weather events and flooding are expected to intensify in the South Pacific. Population growth could also lead to more intensive livestock farming; and urbanization in developing countries is often associated with urban and peri-urban slums where diseases of poverty proliferate. Climate change, flooding, population growth, urbanization, poverty and agricultural intensification are important drivers of zoonotic disease transmission; these factors may independently, or potentially synergistically, lead to enhanced leptospirosis transmission in Fiji and other similar settings.

## Author Summary

Leptospirosis is a bacterial infection transmitted from animals to humans, and many outbreaks are associated with flooding. Globally, leptospirosis is responsible for at least a million cases of severe illness each year, and many deaths. The bacteria are excreted in the urine of infected animals; humans can become infected through direct contact with animals or through contaminated water and soil. In Fiji, two successive cyclones and severe flooding in 2012 resulted in 576 cases and 40 deaths. We conducted this study to improve our understanding of the factors that increase the risk of leptospirosis transmission, so that public health control measures can be improved. Our study found that infection risk is related to many factors including individual demographics and behaviour, contact with animals, living conditions, poverty, and flooding risk. With global climate change, flooding is expected to become a bigger problem in the South Pacific. Population growth could lead to more intensive livestock farming; and urbanization in developing countries is often associated with slums with high risk of infectious diseases. Climate change, flooding, population growth, urbanization, poverty and livestock farming are important factors for leptospirosis transmission; these factors may combine to increase the risk of leptospirosis in Fiji and other Pacific Islands in the future.

## Introduction

Leptospirosis is an emerging infectious disease worldwide, with particularly high incidence reported in the Pacific Islands [1,2]. Humans are infected through direct contact with infected animals, or through contact with water or soil that has been contaminated by urine of infected animals. Disease transmission is strongly driven by environmental factors including high rainfall, flooding, natural disasters, population growth, urbanisation, and poor sanitation and hygiene [2–4]. In addition, infection risk depends on individual behaviour (e.g. swimming in fresh water, working outdoors), and contact with animals including livestock, rodents, pets, & wildlife [2,4]. Risk factors for infections and drivers of outbreaks depend on interactions between humans, animals, and the environment, and vary significantly between locations based on environmental, cultural, and socio-demographic factors [4]. Transmission dynamics are therefore highly complex and variable, and likely to evolve with global environmental change of both natural and anthropogenic environments [2,3].

In Pacific island nations, important risk factors for human leptospirosis include outdoor activities, tropical climate, flooding secondary to extreme weather events, and exposure to livestock [5–8]. Subsistence livestock are commonly kept in backyards, and veterinary expertise is generally limited. In some Pacific Islands, rapid population growth and urbanization exacerbate problems with sanitation, access to clean water, and waste management. Most islands have limited human or financial resources for the management and mitigation of the health impacts of natural disasters and climate change [9,10]. In Fiji, leptospirosis was identified as one of the four priority climate-sensitive diseases of major public health concern [11]. A recent systematic review of the global morbidity and mortality of leptospirosis identified tropical islands as particularly high-risk settings [2]. Apart from the tropical climate and high frequency of extreme weather events [3], factors that could contribute to the high risk of leptospirosis on tropical islands include the low biodiversity and delicate ecosystems that make islands vulnerable to invasive species such as rodents [12]; the outdoor lifestyle and associated intense exposure to the environment; and close contact with subsistence livestock animals [2,4].

Climate change is projected to increase the severity of extreme weather events including increased rainfall and flooding in the Pacific Islands [10], and such events have been associated with increased leptospirosis transmission and outbreaks around the world [3,7,13–16]. In 2012, two successive tropical depressions caused severe flooding and resulted in two outbreaks of leptospirosis in Fiji, with 576 reported cases and 40 deaths (7% case-fatality) (Fiji Ministry of Health and Medical Services [MHMS]). Cases were defined as positive reactions to *Leptospira* ELISA IgM (Panbio, Brisbane, Australia); this laboratory test was only available at the national reference laboratory, and it was likely that reported cases were an underestimate of the true scale of the outbreaks. In comparison, previous studies in Fiji reported a total of 576 cases during an 8-year period from 2000–2007 [17], and 487 cases during a 13-year period from 1969–1981 [18]. These studies identified a higher risk of infection in males, indigenous Fijians (iTaukei), young adults (aged 15 to 45 years), rural dwellers and abattoir workers; increase in reported cases in the rainy months and after a cyclone in 2001; and geographic variation in incidence.

Following the outbreaks in 2012, the Fiji MHMS and the World Health Organization convened a leptospirosis expert consultation to review the epidemiology of leptospirosis in Fiji and recommend priorities for control of endemic and epidemic disease. A key conclusion of the expert consultation was that significant knowledge gaps in the current epidemiology of leptospirosis in Fiji limited effective prevention and control. The study described in this paper was identified as one of several important steps to address the knowledge gaps. This study uses an eco-epidemiological approach and framework [19] to characterize the epidemiology and risk factors for human leptospirosis infection in Fiji, and aimed to provide an evidence base for improving the effectiveness and efficiency of public health mitigation and intervention strategies. Our findings would also be relevant to other countries with similar environments, particularly in the South Pacific.

## Methods

### Study location and population

The Republic of the Fiji Islands is an archipelago of 322 islands with a population of 837,217 in 2007; indigenous Fijians (iTaukei) and Indo-Fijians (Fijians of Indian descent) account for 57% and 35% of the population respectively [20]. Fiji is considered a ‘small island developing state’ by the United Nations [21] with a per capita GDP of US$4,712 [22]. The main island of Viti Levu has a landmass of 10,349 square kilometers and is home to >70% of the population. Vanua Levu is the second largest island in both population and land area, followed by Taveuni. The largest urban centre is the Greater Suva Area (population ~180,000) on the southeast coast of Viti Levu. The largest administrative units in geographical size are Divisions (Central, Western, Northern, and Eastern) followed by Provinces (14 in total), Tikinas (86 in total), and Enumeration Areas (smallest unit for population census that typically include 80 to 120 households). Nursing zones are the smallest administrative unit of the MHMS; they are under the care of a single nursing station and form a contiguous network across the Fijian Islands. Communities are residential clusters used by MHMS for administrative purposes. The four main community types in Fiji are urban residential areas, villages, Indo-Fijian settlements, and mixed Indo-Fijian/iTaukei settlements.

### Seroprevalence study and sampling design

Field data were collected from September to December 2013 (January to March being the wettest months), and included the Central Division (on the eastern side of Viti Levu), the Western Division (on the western side of Viti Levu), and the Northern Division (the islands of Vanua Levu and Taveuni). The Eastern Division, with a population of ~40,000 spread across multiple small islands groups, was not included in the study because of logistical reasons. Field data were collected for a sero-epidemiological study of typhoid as well as the leptospirosis study described here.

**Fig 1:**
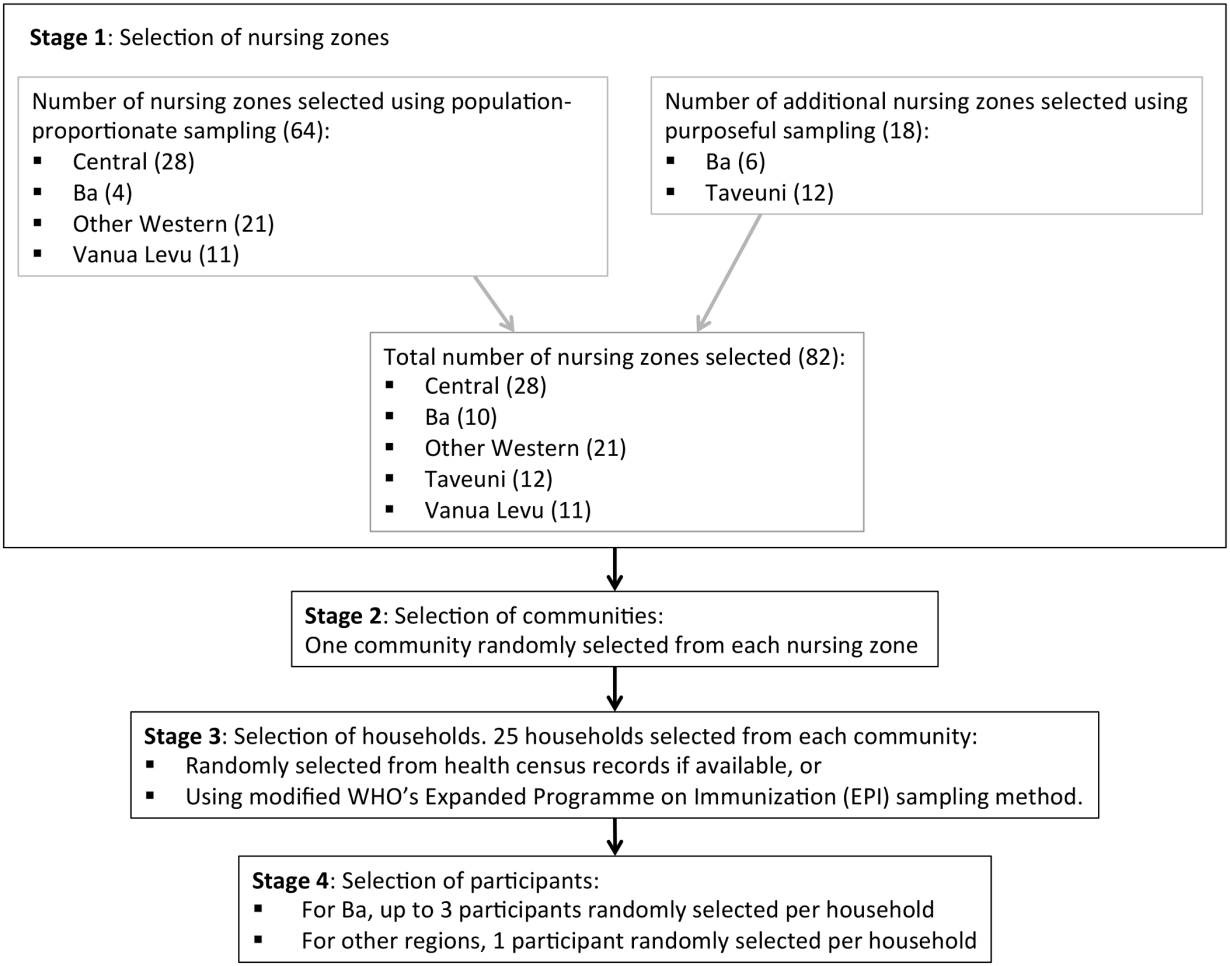
Overview of sampling strategy used in 2015 field study.

We conducted a cross-sectional seroprevalence study, with a four-stage sampling design. An overview of the sampling plan is shown in Fig 1. In the first stage, both population-proportionate sampling and purposeful sampling approaches were used. The former was used to select 28 nursing zones from the Central Division, 21 from the Western Division, 11 from the Northern Division and 4 from the Ba Province which lies within the Western Division. Due to high incidence of reported leptospirosis and post-flood outbreaks in 2012, the latter sampling approach was used to select 6 more nursing zones from the Ba Province. Similar to Ba Province, Taveuni Island (part of the Northern Division) was oversampled because of a high incidence of typhoid in 2008–2009. Consequently, 12 additional nursing zones were selected from this region, resulting in 82 zones in total being selected from the five regions in the first stage of sampling. At the second stage of sampling, one community was randomly selected from each of the 82 nursing zones. Headmen, health workers and other community leaders were consulted to seek agreement to participate in the study; no community leaders declined participation. At the third stage of sampling, 25 households were randomly selected from each community using health census records if available, or using a modified World Health Organization’s Expanded Programme on Immunization (EPI) sampling method. For the fourth and final stage of sampling, household members (defined as a person who stayed at the house the previous night) were enumerated and one selected at random for inclusion. In Ba subdivision, up to three randomly selected household members were included. If a selected household member was absent but returning later that day, the survey team would await their return or made a repeat visit. Wholly absent household members were substituted from within the household. Empty households were substituted by selecting the nearest house to the right of the front door. The above sampling strategy aimed to include 25 households from each of 82 communities, with up to three participants per household in Ba, and one participant per households in other areas. We therefore aimed to recruit a total of 2050 to 2250 participants.

Participants were eligible for inclusion if they were aged 12 months or older. Exclusion criteria included clotting disorders or medical anticoagulation, severe underlying medical conditions, significant acute illness, and fear of needles.

The communities included in the study represented the general population and different environments in Fiji (urban, peri-urban, rural), with higher sampling density in Ba and Taveuni. The study successfully included a total of 81 communities, with 28 in Central Division, 10 in Ba, 21 in other parts of the Western Division, 11 in Taveuni, and 11 in Vanua Levu. These areas will be referred to as the five ‘regions’ in this paper.

### Informed consent and ethics approvals

Ethics approvals were granted by the Fiji National Research Ethics Review Committee (2013 03), the Human Research Ethics Committee of The University of Queensland (2014000008) and the London School of Hygiene & Tropical Medicine (6344). Support was sought and obtained from divisional and sub-divisional Ministry of Health officers for community visits. To ensure that research activities were culturally acceptable and local customs respected, community visits were conducted with field teams that included multilingual local Fijians. The study was explained to the heads of each of the randomly selected households, or another competent adult, and permission sought to include the household in the study. Written or thumb-printed informed consent was obtained from adult participants. The ethics committees specifically approved the use of thumbprint informed consent in illiterate participants. Parental/guardian consent and informed assent was obtained for child participants.

**Fig 2:**
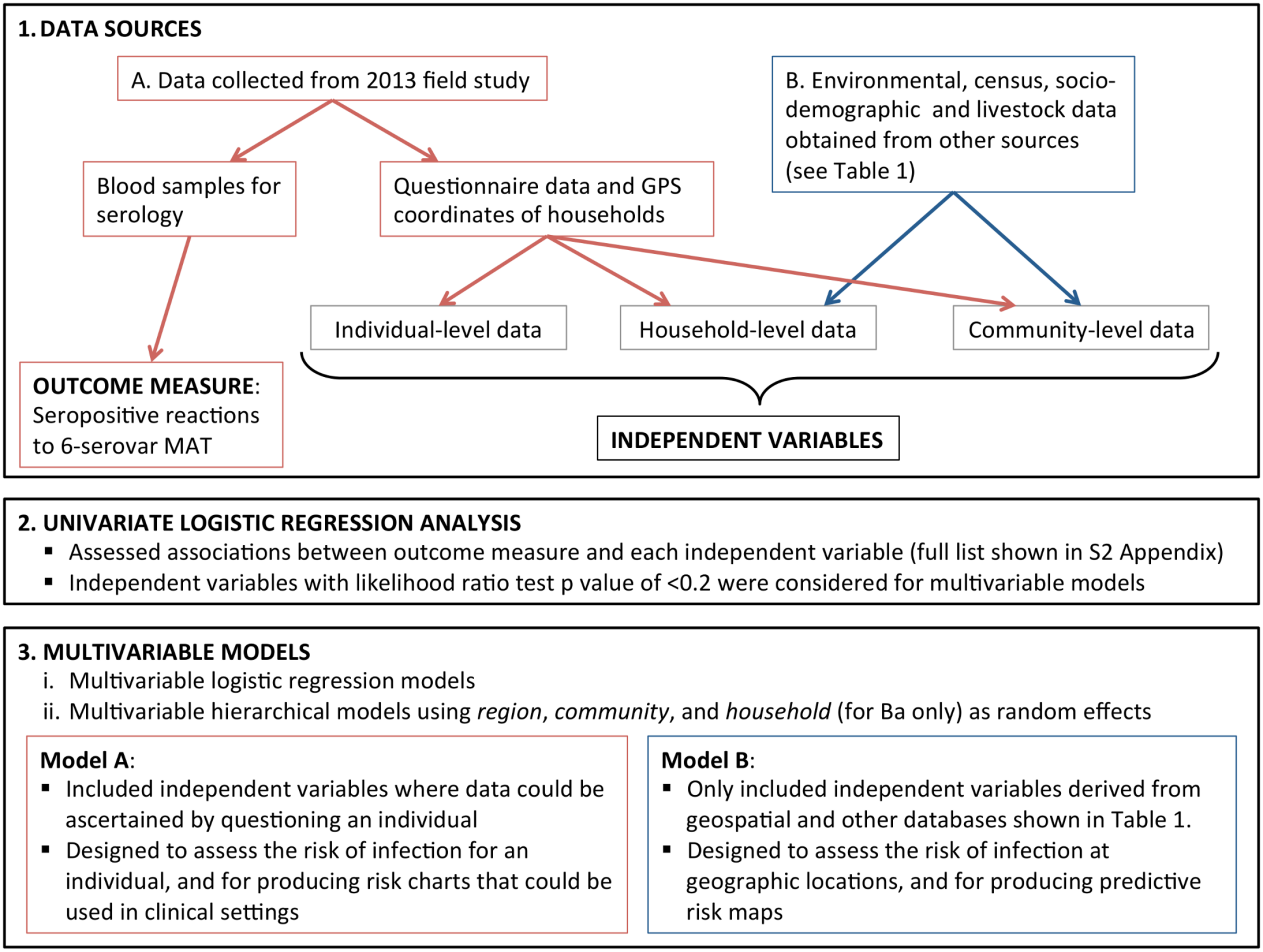
Overview of data sources and statistical methods.

An overview of data sources and statistical methods is shown in Fig 2.

### Data collection during field study

The following were collected from each participant:

1. Venous blood samples, collected by trained phlebotomists under sterile conditions (5–8mL depending on the age of the participant).
2. Questionnaire data, using standard questionnaires administered by field research assistants, and conducted in English or other local languages depending on each participant’s preference. Questions related to demographics, income, occupation, recreational activities, household environment, contact with animals, and other potential risk factors for leptospirosis.
3. Geographic Positioning System (GPS) coordinates of the place of residence, using handheld GPS devices.

### Environmental, census, socio-demographic and livestock data

**Fig 3:**
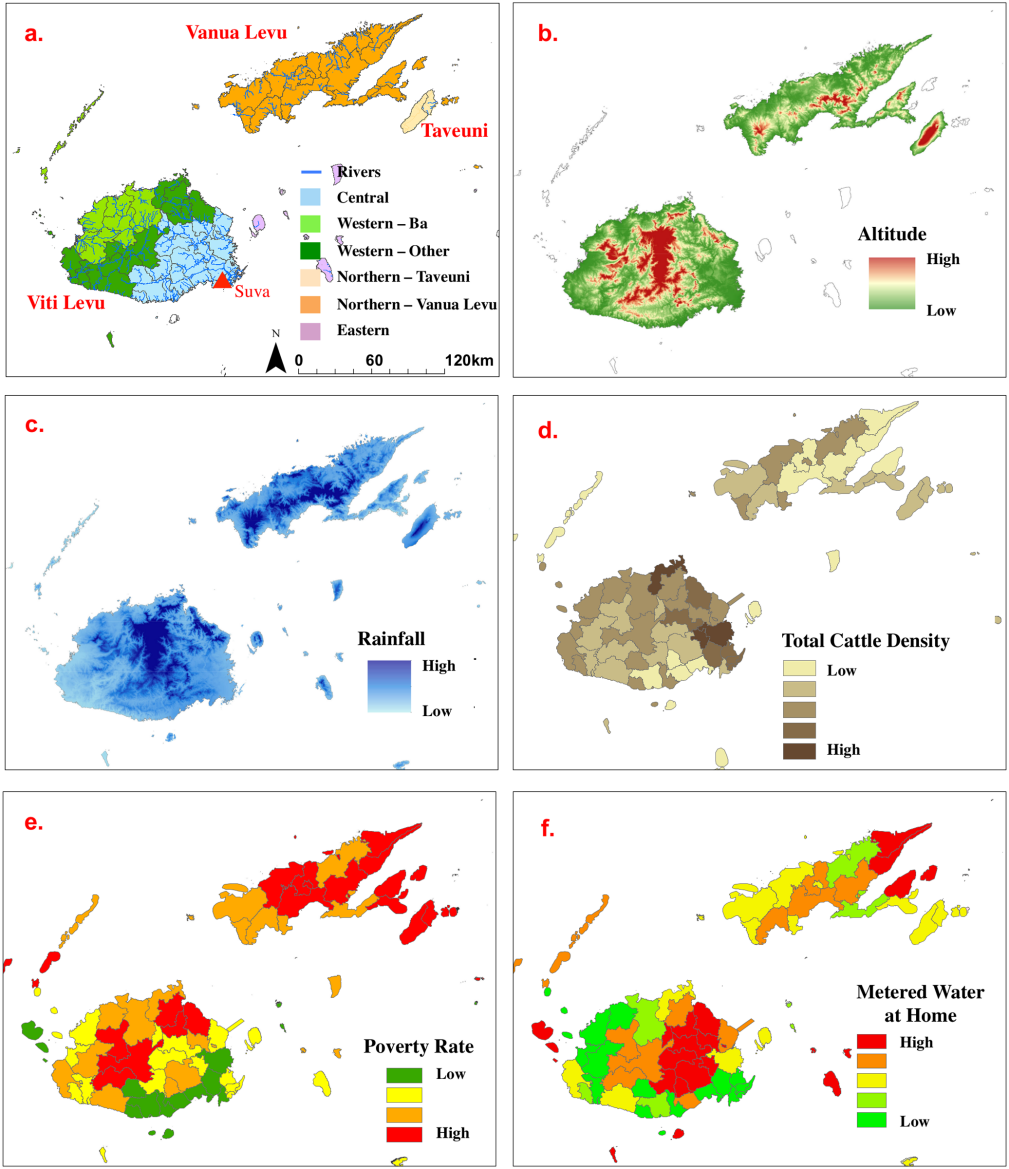
Fiji geography, and examples of environmental and census data used: a) Divisions and ‘regions’ included in the study, major rivers; b) altitude; c) rainfall; d) total cattle density; e) poverty rate; f) proportion of households with metered (treated) water at home. See Table 1 for data sources.

Environmental data on hydrology and roads were obtained from the Fiji Ministry of Lands and Mineral Resources [23]; and soils and land use/cover data from Fiji Ministry of Agriculture [24]. Climate (temperature and rainfall) and elevation data were obtained from the Landcare Research Institute [25]. Data on educational attainment, household construction, employment, ethnicity, and other socio-demographic variables were obtained from the 2007 Fiji National Census [20], and data on poverty rates from the 2011 World Bank Report [26]. Livestock data were provided by the Fiji Ministry of Agriculture’s 2009 National Agricultural Census [27]. All geospatial data were georeferenced to the Fiji Map Grid 1986 coordinate system. Table 1 provides a summary of the environmental, census, socio-demographic and livestock data used in the study. The five geographic regions used in this study and examples of the geo-referenced data are shown in Fig 3.

**Table 1:** Summary of environmental, census, socio-demographic and livestock data used.

Data	Source	Variables examined	Description & Resolution
**Hydrology**	Fiji Ministry of Lands and Mineral Resources. Digital data from FLIS (Fiji Land Information System). Original 1:50K topographic maps [23].	Distance to rivers, major, minor creeks.	Euclidean distance to rivers, major, minor creeks. 25 m raster data.
**Roads**	Fiji Ministry of Lands and Resources. Digital data from FLIS (Fiji Land Information System). Original 1:50K topographic maps.	Road density.	Number of roads per sq km within 1 km radius. 25 m raster data.
**Soils**	Fiji Ministry of Agriculture. 1980/85 National Soil Survey [24].	Soils of major and secondary floodplains, and depressions.	Soil units and distance from soil units. 25 m raster data.
**Land use/cover**	Ministry of Agriculture. Digital data from Secretariat of the Pacific (SPC). Circa 2010.	Multiple land use/cover types.	Visual interpretation of satellite imagery. 25 m raster data.
**Climate**	Landcare Research Institute [25].	Annual, maximum, minimum, and average temperature and rainfall	Spatially interpolated climate data meteorological station data from 1971–2000. 100 m raster data.
**Elevation**	Landcare Research Institute [3].	Altitude and slope	Elevation derived from 20 m contours. 25 m raster data.
**Census**	Fiji National Census 2007 [20].	Multiple measures of education attainment, house construction, employment, ethnicity, and other sociodemographic factors.	Census variables available at the Enumeration Area level (~80–120 households). Vector count data converted to proportions.
**Economic status**	World Bank (2011) report [26].	Poverty rate (% of population below poverty line) and poverty gap (how far on average. people are from the poverty line)	Poverty rates estimated using small area estimation method. Vector data at the Tikina level.
**Livestock**	Fiji Ministry of Agriculture. Fiji National Agricultural Census 2009 [27].	Number of farms and farm animals by species: cattle, commercial beef, dairy, subsistence beef, etc.	Density per sq km calculated by Tikina (not available for all Tikinas).

Household GPS coordinates from the study were projected on to the Fiji Map Grid 1986 coordinate system. Attributes from the geospatial predictor layers were linked to each household location by intersecting points through polygons for vector GIS data, and sampling the raster GIS data in a similar fashion. As a result, GIS attributes for each predictor layer were obtained for each household location. Attributes for a community were obtained by first calculating the location of the median centre of sampled households, followed by an approach similar to that which was carried out for individual households. All GIS data preparation and analysis was performed using ArcGIS v10.1 (Environmental Systems Research Institute, Redlands, CA).

All GIS data preparation and analysis was performed using ArcGIS v10.1 (Environmental Systems Research Institute, Redlands, CA).

### Stratification of independent variables

Independent variables were stratified according to the ecological level at which they could potentially influence the risk of leptospirosis transmission and infection. Individual-level data relate to risk factors that are specific to individual demographics or behaviour. Household-level and community-level data include risk factors that are common to all inhabitants of a household and community respectively.

- **Individual-level data**. Potential risk factors for leptospirosis were assessed using questionnaire-based interviews, including demographics, occupation, recreational activities, contact with animals, education, and knowledge about leptospirosis.
- **Household-level data**. Information on household income, house construction, access to utilities (toilets, water, sewage, electricity), and the presence of animals and crops around the home were obtained through questionnaires. In addition, data on environmental attributes (including rainfall, temperature, elevation, land cover, soil type, and distance to rivers) at household locations were extracted or calculated using geographic information systems (GIS) as described above.
- **Community-level data**. Community type, urban/rural settings, and the presence of animal species in each community were ascertained through questionnaires. Census and agricultural data were extracted or calculated using the geospatial databases described in Table 1. Data were available at the enumeration area resolution (~80–120 households) for a variety of socioeconomic and demographic measures, including the proportions of households with metered water, toilets, electricity, and sewage services; population ethnicity, level of educational attainment, and reliance on subsistence farming as the main source of income. At the Tikina level, data were available on World Bank estimates of poverty measures, and census of animal populations conducted by the Fiji Ministry of Agriculture.

### Maps

Maps were produced to show the locations of communities that participated in the field study, and the observed community-level seroprevalence in 2013. Although all household GPS locations were recorded, only community-level seroprevalence were depicted on maps to protect the identity of participants. Locations of communities were mapped to their median centre, calculated as the location nearest to all sampled households in the community while minimizing the effects of outliers.

### Serological analysis

Blood samples were processed in Fiji, and frozen sera transported to Australia for serological analysis. Microscopic agglutination tests (MAT) were used to detect anti-*Leptospira* antibodies, and determine the putative serogroups associated with infections. The MAT is the reference serological test recommended by the WHO and the International Committee on Systematic Bacteriology (Subcommittee on the Taxonomy of *Leptospira*) [28,29]. Serological analyses were conducted at the WHO/FAO/OIE Collaborating Centre for Reference & Research on Leptospirosis in Brisbane, Australia. Based on the laboratory’s knowledge of the epidemiology of leptospiral serovars in the South Pacific, 21 pathogenic serovars (see S1 Appendix) were selected for the initial MAT panel for this study, and samples were tested at dilutions from 1:50 to 1:3200. The 21-serovar panel was used to test a random selection of ~10% of total samples to determine the most common serogroups responsible for infections. In addition, the 21-serovar panel was used to test 199 *Leptospira* ELISA-positive samples collected from patients with suspected clinical leptospirosis in Fiji in 2012 and 2013 to ensure that the most common serogroups associated with clinical infections were included in the final panel. Based on the MAT results from the two sets of sera, six serovars were chosen for the final panel used to test the remaining samples from this study (S1 Appendix): *Leptospira interrogans* serovars Pohnpei (serogroup Australis), Australis (serogroup Australis), Canicola (serogroup Canicola), Copenhageni (serogroup Icterohaemorrhagiae), Hardjo (serogroup Sejroe), and *Leptospira borgpetersenii* serovar Ballum (serogroup Ballum).

The MAT assay is expensive and time-consuming, and the described strategy to limit the number of serovars included in the final panel resulted in reduced project costs. Considering that one dominant serovar was identified in the preliminary tests, it was determined that the smaller MAT panel was unlikely to have significant impact on the overall epidemiological findings. MAT titres of ≥1:50 were considered reactive or seropositive, and indicative of recent or past infection. For samples that reacted to multiple serovars within a serogroup, the serovar associated with the highest titre was considered to be the reacting serovar. Samples that reacted to serovars in more than one serogroup were recorded as reacting to multiple serovars. Although serogroups are no longer used in the taxonomic classification of serovars, they remain useful for laboratory purposes and epidemiological comparisons.

### Statistical analysis

An overview of the statistical analyses is shown in Fig 2. The outcome measure used was seropositive reactions to any of the six serovars included in the final MAT panel. Firstly, crude associations between the independent variables and the outcome measure were obtained by univariable logistic regression. Independent variables associated with the outcome by a likelihood ratio test (LRT) p-value of <0.2 were then subjected to a stepwise backward elimination process (p<0.05) to select the final set of independent variables for the multivariable logistic regression models. In addition, the possible presence of effect modification in the multivariable modelling was investigated using the LRT. This was assessed using interaction terms, which consisted of all independent variables found to be significant in the univariable analysis. Interaction terms were added separately to the analyses to determine their joint effect on the outcome measure. Multilevel hierarchical modelling was used to take into account the clustering of participants, and allowed for correlation of observations by *region* (n = 5), *community* (n = 81), and *household* (up to 3 participants per household in Ba) as random effects. Intra-cluster correlation coefficients (ICCs) with corresponding 95% confidence intervals were obtained from final multivariable models. Biological plausibility and collinearity between variables were taken into account when selecting the variables to be retained in the final models. For example, if we observed strong collinearity between poverty rate and % of households in the community with electricity supply, poverty rate would be chosen for the final model because of more direct relationship to exposure risks.

Two multivariable models were built:

- **Model A**. Used independent variables where data could be ascertained by questioning an individual, and included primarily individual-level variables, but also some household-level and community-level variables. Model A was designed to assess the risk of infection for an individual, e.g. for producing predictive risk charts to graphically depict the combined effects of variables in determining overall seroprevalence. The charts are designed for use in clinical settings, and are similar to cardiovascular risk charts used to predict the risk of cardiac events based on combinations of risk factors such as blood pressure, diabetes, smoking, and cholesterol levels.
- **Model B**. Used independent variables derived from geospatial and other national databases described in Table 1, without using the field data collected in this study. Model B included household-level and community-level variables only, and was designed to assess the risk of infection at geographic locations, e.g. estimating community-level seroprevalence, identifying hotspots and producing predictive risk maps.

Independent variables found to be statistically significant on multivariable regression analyses were reported. Adjusted odds ratios (OR) with 95% confidence intervals obtained from regression coefficients were used to quantify associations between the independent and outcome variables. In addition, univariate results of variables associated with animal exposure and contact were reported. Statistical significance was considered at *p* < 0.05 and two-sided. Data analysis was performed using STATA 13 (StataCorp, 2013). Model fit was assessed using the Hosmer-Lemeshow test [30], while relative predictive performance was undertaken using the area under the receiver operating curve (AUC) was calculated for each model and compared for statistical differences. An AUC of 0.7 was deemed to indicate an adequate predictive ability of the model. Akaike information criterion (AIC) and Bayesian information criterion (BIC) were reported for the final models.

## Results

### Study population

**Fig 4:**
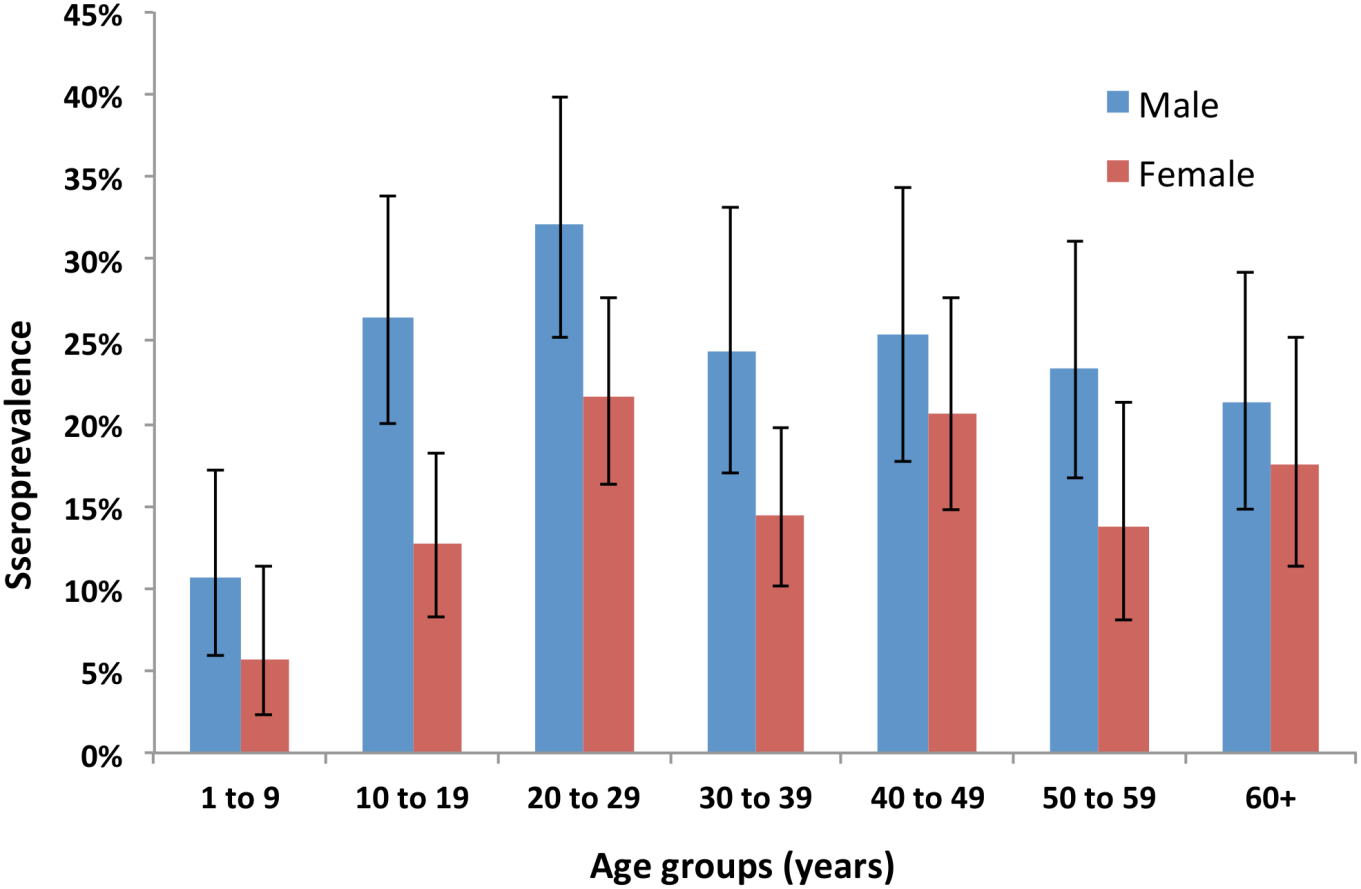
Seroprevalence by age group and gender. Seroprevalence was defined as the percentage of participants with reactive MAT (≥ 1:50) to at least one of the 6 serovars used in the final panel. Blue = male. Red = female.

A total of 2152 participants from 1922 households in 81 communities on the three main islands of Fiji were included in the study. The age of participants ranged from 1 to 90 years (mean 33.6, SD 19.8), and 985 (45.8%) were males. The age and sex distribution of participants are shown in Fig 4. The study included 662 participants from the Central Division (28 communities), 453 from Ba (10 communities), 520 from other parts of the Western Division (21 communities), 261 from Taveuni (11 communities), and 256 from Vanua Levu (11 communities) (Table 2). One of the selected communities in Taveuni was not included because of logistic constraints.

**Table 2:** *Leptospira* seroprevalence by age, gender, ethnicity, community types, and region.

Variables	No of participants	Reactive MATs*	Sero-prevalence (%)	95% CI
**Total sampled**	2152	417	19.4%	17.7–21.1%
**Gender**				
Male	985	234	23.8%	21.1–26.5%
Female	1160	182	15.7%	13.6–17.9%
**Age groups (years)**				
0–9	256	21	8.2%	5.1–12.3%
10–19	362	69	19.1%	15.1–23.5%
20–29	387	101	26.1%	21.8–30.8%
30–39	340	61	17.9%	14.0–22.4%
40–49	279	63	22.6%	17.8–27.9%
50–59	263	50	19.0%	14.5–24.3%
≥ 60	263	52	19.8%	15.1–25.1%
**Ethnicity**				
Indo-Fijian	459	34	7.4%	5.2–10.2%
iTaukei	1651	374	22.7%	20.7–24.7%
Other	39	8	20.5%	9.3–36.5%
**Community type**				
Private residential	502	44	8.8%	6.4–11.6%
Settlement (Indo-Fijian)	103	18	17.5%	10.7–26.2%
Settlement (mixed ethnicity)	511	91	17.8%	14.6–21.4%
Village	1036	264	25.5%	22.9–28.3%
**Urban/Rural**				
Urban	579	64	11.1%	8.6–13.9%
Peri-urban	287	44	15.3%	11.4–20.0%
Rural	1286	309	24.0%	21.7–26.5%
**Region**				
Central Division	662	107	16.2%	13.4–19.2%
Western Division–Ba	453	82	18.1%	14.7–22.0%
Western Division–Other	520	94	18.1%	14.9–21.7%
Northern Division–Taveuni	261	59	22.6%	17.7–28.2%
Northern Division–Vanua Levu	256	75	29.3%	23.8–35.3%

*Reactive MAT defined at titre of ≥1:50 for one or more serovars used in the 6-serovar panel

### Seroprevalence and serovars

**Fig 5:**
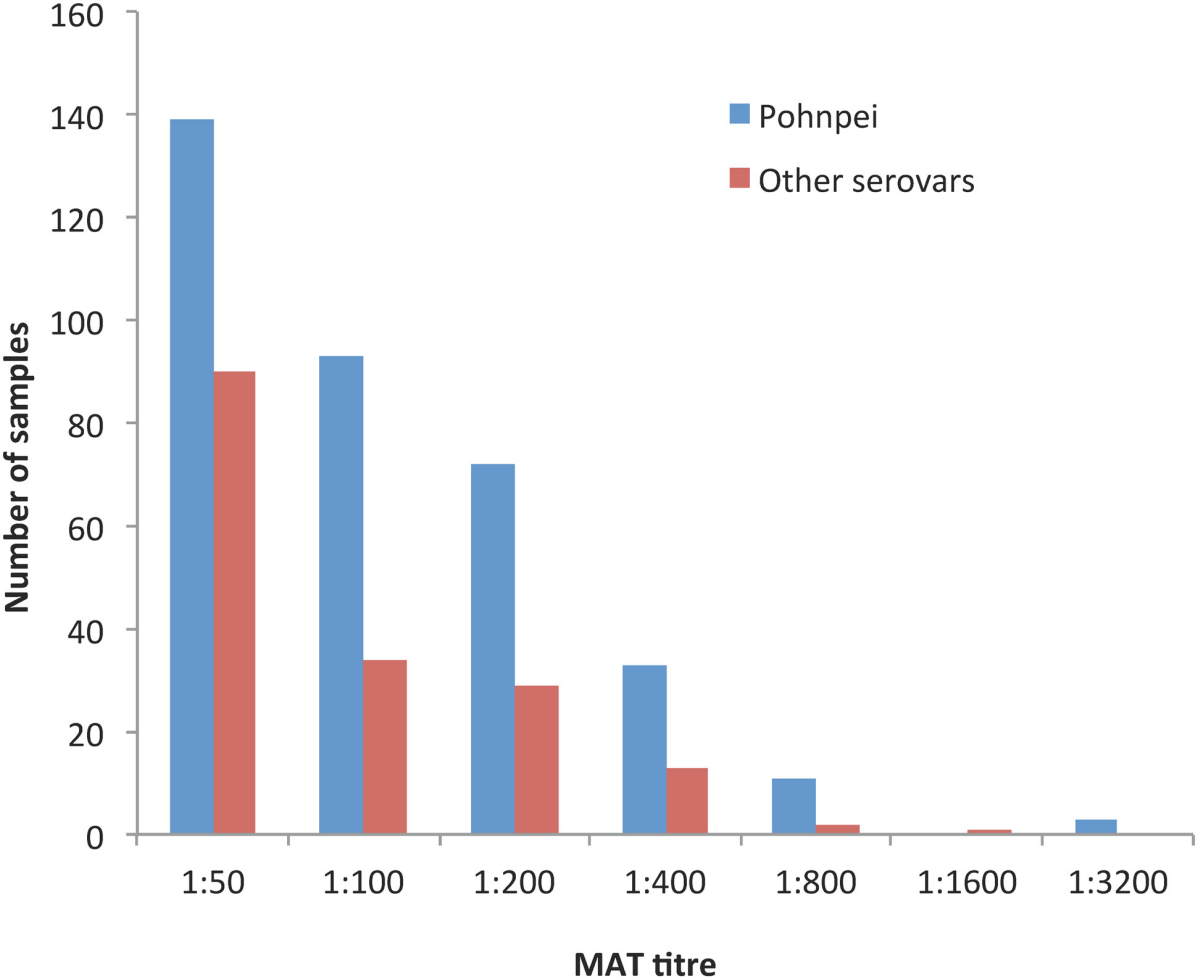
Distribution of MAT titres for serovar Pohnpei (blue) and other serovars (red); using the final panel of 6 serovars.

Details of the 21 serovars included in the screening panel and the 6 serovars included in the final panel are shown in S1 Appendix, together with the seroprevalence of the initial 198 randomly selected samples from this study and the 199 *Leptospira* ELISA-positive samples from patients with suspected leptospirosis from April 2012 to November 2013., The six serovars included in the final MAT panel accounted for 86.7% of reactive tests: *Leptospira interrogans* serovars Pohnpei, Australis, Canicola, Copenhageni, and Hardjo; and *Leptospira borgpetersenii* serovar Ballum. Using the 6-serovar panel, the overall seroprevalence was 19.4% (95% CI 17.7%–21.1%), with 417 participants having reactive MATs to at least one serovar. One predominant serovar, Pohnpei, accounted for 351 (84.2%; 95% CI 80.3%–87.5%) of reactive MATs. A total of 63 participants had MAT titres of ≥1:400 (47 for serovar Pohnpei, and 16 for other serovars), the cutoff used by our laboratory to indicate an acute infection. The distribution of MAT titres for Pohnpei and other serovars is shown in Fig 5.

**Fig 6:**
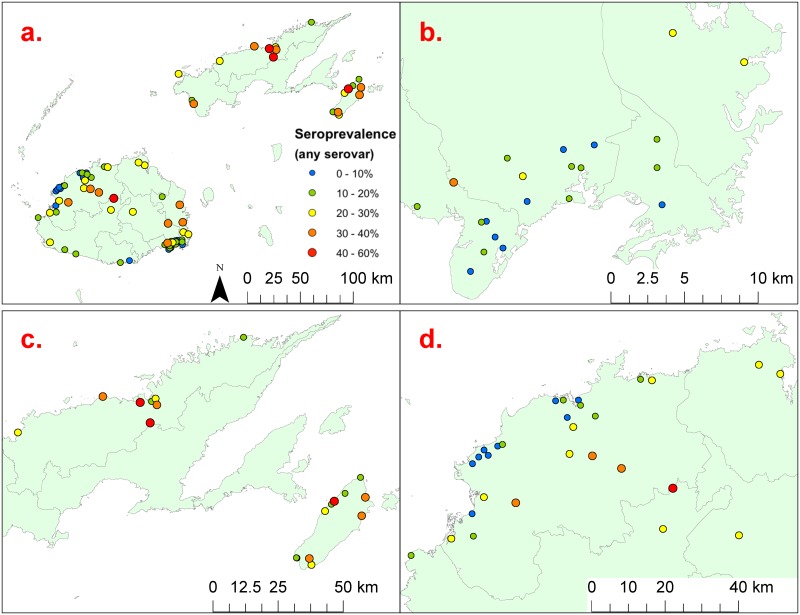
Community-level seroprevalence at the 81 communities included in the study; a) prevalence varied from 0% to 60%; b) enlargement of the Suva area in eastern Viti Levu; c) enlargement of Taveuni and eastern Vanua Levu; and d) enlargement of northwestern Viti Levu including Ba.

**Fig 7:**
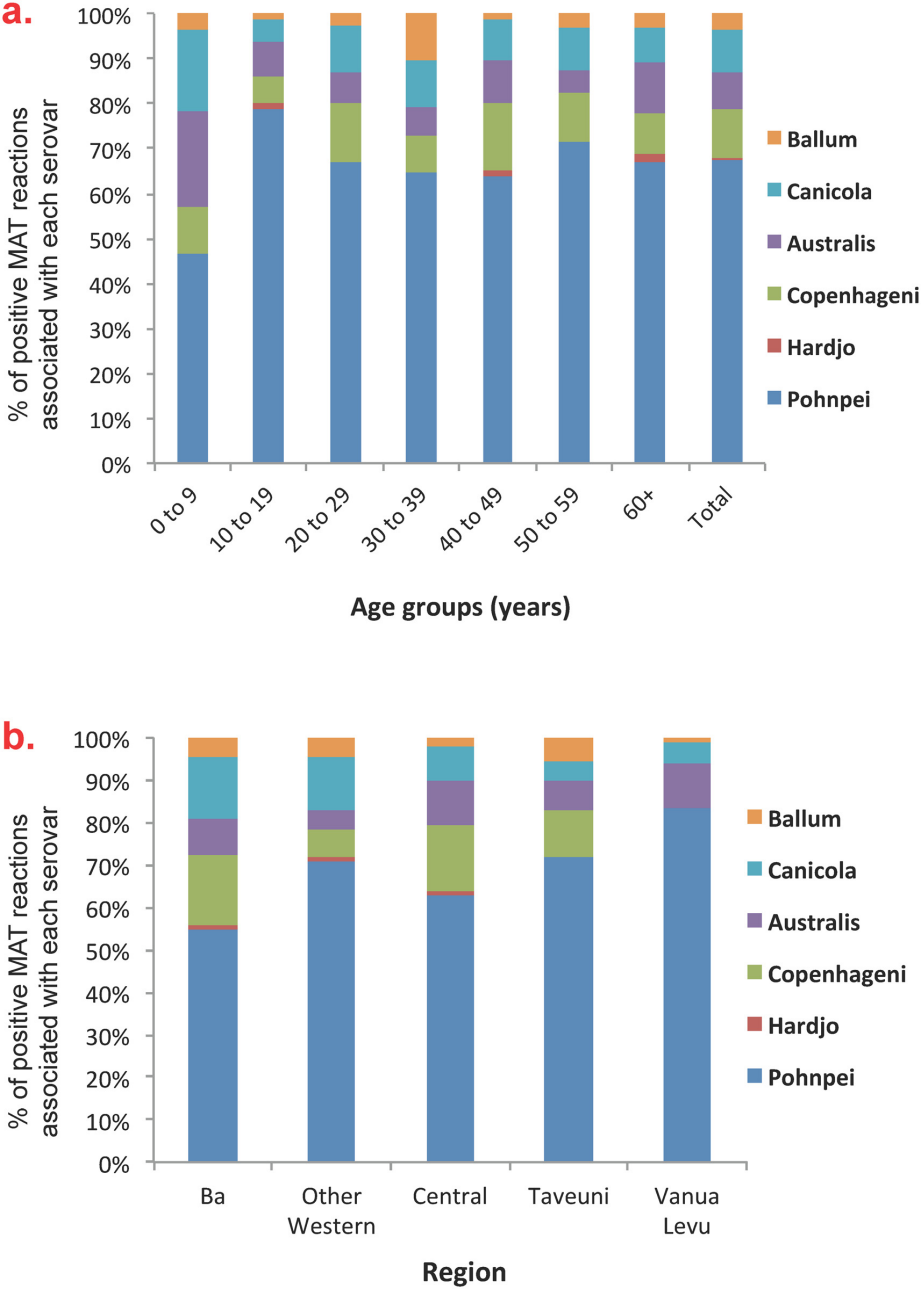
Percentage of positive MAT reactions associated with each of the 6 serovars included in the final panel by: a) age groups, and b) regions. Positive MAT reactions defined as titre of ≥ 1:50.

Table 2 shows that there were significant differences in seroprevalence by age, gender, ethnicity, community types, and region. Community-level seroprevalence ranged from 0% to 60%, and are shown on the maps in Fig 6a to 6d. Variations in seropositive reactions to each serovar by age groups and region of residence are shown in Fig 7a & 7b respectively.

### Risk factor analysis and multivariable models

A total of 118 independent variables were assessed on univariate analysis: 75 variables obtained from questionnaires, and 43 derived using GIS from the sources described in Table 1. Independent variables included 31 individual-level, 38 household-level, and 49 community-level risk factors described above. S2 Appendix provides a list of the independent variables assessed at the univariate level. Variables statistically significant on univariate analyses were considered for the multivariable models, and included 19 individual-level, 21 household-level, and 25 community-level risk factors. Due to statistical significance not be reached, no interaction effect was included in the multivariable modelling.

Multivariable Model A (using variables where data could be ascertained by questioning an individual) included five variables that were independently associated with the presence of *Leptospira* antibodies, with an AUC of 0.7 (Table 3) including: male gender (OR 1.55 compared to females), iTaukei ethnicity (OR 3.51 compared to Indo-Fijians), living in settlements and villages (OR 2.13 and 1.64 respectively compared to urban residential areas), not having metered water at home (OR 1.52), and working outdoors (OR 1.64 compared to working indoors). Of the 434 participants who worked outdoors, 378 (87%) were full- or part-time farmers, indicating that outdoor work in Fiji is predominantly related to farming.

**Table 3:** Variables significantly associated with positive MAT for *Leptospira* on univariable and multivariable analysis–Model A^ (individual-level variables).

Variables	No of subjects	Reactive MAT*	Sero-prevalence (%)	Univariable Odds Ratio (95% CI)	Adjusted Odds Ratio (95% CI)	*p* value^#^
**Total sampled**	2152	417	19.4%			
**Gender**						
Female	1160	182	15.7%	1	1	
Male	985	234	23.8%	1.67 (1.35–2.08)	1.55 (1.16–2.08)	0.003
**Ethnic group**						
Indo-Fijian	459	34	7.4%	1	1	
iTaukei	1651	374	22.7%	3.66 (2.53–5.29)	3.51 (2.23–5.54)	<0.001
Other	39	8	20.5%	3.23 (1.38–7.56)	2.32 (0.82–6.58)	0.114
**Community type**						
Urban residential	502	44	8.8%	1	1	
Settlement	614	109	17.8%	2.25 (1.55–3.26)	2.13 (1.41–3.21)	<0.001
Village	1036	264	25.5%	3.56 (2.54–5.00)	1.64 (1.08–2.51)	0.021
**Metered water available at home**						
Yes	1412	221	15.7%	1	1	
No	720	189	26.3%	1.92 (1.54–2.39)	1.52 (1.14–2.03)	0.004
**Work location**						
Indoors	832	106	12.7%	1	1	
Mixed indoors/ outdoors	639	123	19.2%	1.63 (1.23–2.17)	1.65 (1.23–2.20)	0.001
Outdoors	434	119	27.4%	2.59 (1.93–3.47)	1.64 (1.15–2.34)	0.006

^ Model goodness of fit: AIC 1675.9, BIC 1731.3, df 10.

*Reactive MAT defined at titre of ≥1:50 for one or more serovars used in the 6-serovar panel

^*#*^ *p* value for adjusted odds ratios, multivariable model

Multivariable Model B (using only variables derived from geospatial and other national databases) included six variables that were independently associated with the presence of *Leptospira* antibodies, with an AUC of 0.7 (Table 4) including: living in rural areas (OR 1.43 compared to living in urban or peri-urban areas), poverty rate ≥ 40% (OR 1.74), living <100m from a river or major creek (OR 1.41), presence of pigs in the community (OR 1.54), total cattle density in the Tikina (OR 1.04 per head of cattle per square km), and high maximum rainfall in the wettest month (OR 1.003 per mm of rain). Total cattle density (includes both commercial and subsistence livestock) ranged from 0.11 to 31.48 head of cattle per square km (mean 8.96, SD 5.31), and maximum rainfall in the wettest month ranged from 275 to 789mm (mean 375.02, SD 56.94). A similar multivariable model using total dairy farm density instead of total cattle density performed better than the final model, but data on dairy farm density were only available for 57 of the 81 (70.4%) communities included in our study, and this variable was therefore not selected for the final Model B.

**Table 4:** Variables significantly associated with positive MAT for *Leptospira* on univariable and multivariable analyses–Model B^ (environmental and census variables).

Variables	No of subjects	Reactive MAT*	Sero-prevalence (%)	Univariable Odds Ratio (95% CI)	Adjusted Odds Ratio (95% CI)	*p* value^#^
**Urban/Rural**						
Urban/ Peri-urban	866	108	12.5%	1	1	
Rural	1286	309	24.0%	2.22 (1.75–2.82)	1.43 (1.07–1.91)	0.016
**Poverty rate**						
< 40%	1277	187	14.6%	1	1	
≥ 40%	875	230	26.3%	2.08 (1.67–2.58)	1.74 (1.31–2.31)	<0.001
**Distance between home and river or major creek**						
> 100m	1590	279	17.6%	1	1	
≤ 100m	456	115	25.2%	1.58 (1.24–2.03)	1.41 (1.09–1.83)	0.009
**Presence of pigs in community**						
No	1587	266	16.8%	1	1	
Yes	561	150	26.7%	1.81 (1.44–2.28)	1.54 (1.21–1.98)	0.001
	Mean (Standard deviation)					
**Total cattle density in Tikina^§^ (per head of cattle per sq km)**						
Seronegative subjects	8.86 (5.18)					
Seropositive subjects	9.38 (5.83)			1.02 (1.00–1.04)	1.04 (1.02–1.06)	<0.001
**Maximum rainfall in wettest month (per mm)**						
Seronegative subjects	372.83 (50.05)					
Seropositive subjects	384.18 (79.01)			1.003 (1.001–1.005)	1.003 (1.001–1.005)	0.002

^Model goodness of fit: AIC 1924.3, BIC 1963.6, df 7.

*Reactive MAT defined at titre of ≥1:50 for one or more serovars used in the 6-serovar panel

^*#*^ *p* value for adjusted odds ratios, multivariable model

^§^ Includes both commercial and subsistence cattle

Collection of biological samples from animals was outside of the scope of this study, but study questionnaires included detailed information about contact with animals (rodents, mongoose, pets, and livestock) at home and the presence of animals in the community. A number of animal-related exposures were significantly associated with the presence of *Leptospira* antibodies on univariable analysis (Table 5). The presence of rats, mice, and mongoose around the home was not significantly associated with seroprevalence, but higher infection rates were found in participants who reported physical contact with rats or mice (OR 1.58) and mongoose (OR 1.81). Table 5 shows that many Fijians have livestock animals at home and in the community. The presence of each livestock species was associated with a higher infection rates on univariable analysis, but only the presence of pigs in the community was significant on multivariable analysis, and included in Model B.

**Table 5:** Associations between positive MAT for *Leptospira* and animal exposure at home and in the community.

Questions related to animal exposure and contact	Number of subjects who answered ‘yes’	% of subjects who answered ‘yes’	Univariable Odds Ratio (95% CI)	*p* value
Seen rats or mice at or around your home	1844	85.9%	1.16 (0.84–1.59)	0.371
Been in physical contact with rats or mice	323	15.3%	1.58 (1.20–2.09)	0.001
Seen mongooses at or around your home	1655	77.1%	1.08 (0.83–1.39)	0.574
Been in physical contact with mongooses	135	6.5%	1.81 (1.23–2.68)	0.003
Pigs at your home or in your garden	230	10.7%	1.55 (1.23–2.12)	0.007
Pigs in your community	561	26.1%	1.81 (1.44–2.28)	0.000
Cows at your home or in your garden	284	13.2%	1.53 (1.15–2.05)	0.004
Cows in your community	481	22.4%	1.52 (1.19–1.93)	0.001
Horses at your home or in your garden	200	9.3%	1.53 (1.09–2.14)	0.013
Horses in your community	377	17.6%	1.55 (1.19–2.01)	0.001
Are there goats at your home or in your garden?	107	5.0%	1.08 (0.67–1.75)	0.749
Goats in your community	242	11.3%	1.47 (1.08–2.01)	0.015
Chickens at your home or in your garden	431	20.1%	1.21 (0.93–1.57)	0.152
Chickens in your community	819	38.1%	1.39 (1.12–1.72)	0.003
Dogs at your home or in your garden	645	30.0%	1.00 (0.79–1.26)	0.992
Dogs in your community	998	46.5%	1.25 (1.01–1.55)	0.041
Cats at your home or in your garden	355	16.5%	0.78 (0.58–1.06)	0.115
Cats in your community	830	38.6%	1.38 (1.11–1.72)	0.003

Multilevel hierarchical models were built to take into account spatial correlation of data: i) defining *region* and *community* as random effects, using the entire dataset, and ii) defining *community* and *household* as random effects, using Ba data only. The results of multilevel models were not statistically different to the results of multivariable Model A using all data (chi2 = 0.01, *p* = 1.00), Model A using Ba data (chi2 = 0.00, *p* = 1.00), Model B using all data (chi2 = 0.01, *p* = 0.99), or Model B using Ba data (chi2 = 0.00, *p* = 1.00). For all multilevel models, intra-class correlation coefficients were <0.01 and odds ratio estimates were very similar to the reported models. Results for the multilevel models were therefore not reported here.

### Seroprevalence estimation chart using Model A

**Fig 8:**
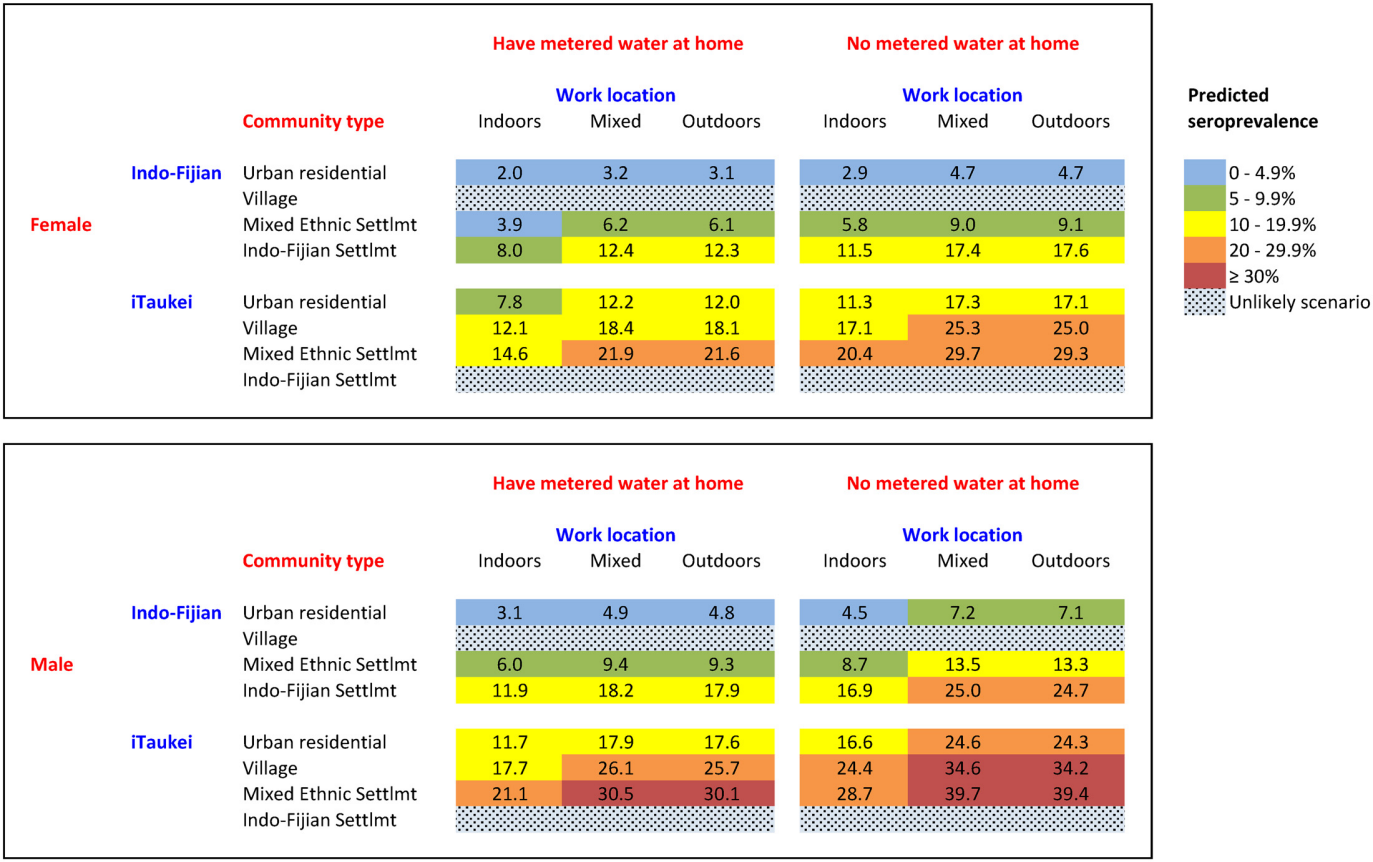
Seroprevalence estimation chart based on Model A, a multivariable logistic regression model of individual-level variables for a) females and b) males. The chart shows the combined effects of independent risk on the estimated prevalence of leptospirosis infection. Seroprevalence was defined as as the percentage of participants with reactive MAT (≥ 1:50) to at least one of the 6 serovars in the final panel.

Fig 8 shows a seroprevalence estimation chart that incorporates individual-level variables to show the combined effects of multiple independent risk factors on the prevalence of infection. Estimated seroprevalence were based on the five variables used in Model A. For example, the chart shows a range of seroprevalence from 2.0% for female Indo-Fijians who live in urban residential areas, have metered water at home, and work indoors; to 39.4% for male iTaukei who live in mixed ethnic settlements, do not have metered water and home, and work outdoors. It is uncommon for Indo-Fijians to live in villages or for iTaukei to live in Indo-Fijian settlements, and results were therefore not shown for these scenarios.

## Discussion

Our study identified a high risk of human leptospirosis infection in Fiji, with an overall seroprevalence of 19.4% using a 6-serovar MAT panel. One dominant serovar, Pohnpei, was associated with 84.2% of reactive MATs. The serovar was originally isolated from rodents and pigs during an animal leptospirosis study in the island of Pohnpei in the Federated States of Micronesia [31], and has been found to be an important cause of human infections [32]. Seroprevalence varied significantly between the five regions in our study, and ranged from 16.2% in the Central Division to 29.3% in Vanua Levu in the Northern Division. Community-level seroprevalence also varied significantly from 0% to 60% in the 81 communities included in our study. These findings indicate marked geographic variation in infection risk in Fiji and the presence of hotspots where disease transmission is more intense.

Globally, reported leptospirosis seroprevalence vary significantly between and within countries, based on environmental settings, behavioural risk factors, and socio-demographics; our results corroborate these findings. To put the Fiji results into a global context, examples of seroprevalence reported from known high risk settings [2,4] such as urban slums, tropical islands, and flood risk areas include 15.4% in an urban slum in Brazil [33], 37% of healthy adult males in the Seychelles [34], 18.8% in the Mekong delta in Vietnam [35], and 23.9% and 38.2% in flood-prone areas in Laos and Bangladesh respectively [36,37]. As found in Fiji, small-scale variations in seroprevalence within countries and differences between occupational groups have been reported. In American Samoa (a group of remote islands in the south Pacific), a community-based study reported an overall seroprevalence of 15.5% [8] and significant variation between islands with different environments, and between areas with different population density [38]. In Peru, seroprevalence varied from 28.0% in the Amazonian city of Iquitos and 16.5% in the surrounding villages (wet tropics), to 0.7% in a desert shantytown near Lima [39]. In the Andaman Islands, a study of high-risk populations found seroprevalence of 62.5% in agricultural workers, 39.4% in sewage workers, 37.5% in animal handlers, and 30.0% in butchers [40]. In contrast, a study of healthy blood donors in an area of high leptospirosis incidence in northern Queensland in Australia found a seroprevalence of only 1.4% [41,42].

Our study found that individual-level factors were important predictors of leptospirosis infection risk in Fiji. Model A shows that gender, ethnicity, community type, availability of water at home, and work location were independently associated with the presence of *Leptospira* antibodies. Higher infection rates in males corroborates findings in the majority of leptospirosis studies around the world, and is likely to be associated with higher frequency of outdoor activities as well as higher risk occupational and recreational exposures. Reasons for the marked difference in seroprevalence between the two main ethnic groups in Fiji are unclear, but could be related to differences in genetic susceptibility or behaviours that were not elucidated by our questionnaire, e.g. differences in animal husbandry or slaughtering practices related to religion or culture. Further studies are required to explain the disparate risk between ethnic groups. Seroprevalence in villages was significantly higher than in urban residential areas or settlements, and is likely the result of more intimate contact with the natural environment and domestic animals. In our study, working outdoors was associated with a higher risk of infection, and the majority of outdoor work in Fiji involves farming. Agriculture is an important part of Fiji’s economy, and apart from the livestock industry, there is commercial farming of a range of crops include sugarcane, coconut, copra, and a wide variety of fruits and vegetables. Occupational exposure in the agricultural industry is therefore likely to be an important source of leptospirosis infection in Fiji.

Of note, three of the predictors included in our final multivariable models were related to water: the *availability of metered (treated) water at home* (Model A), *distance between the home and the closest river or major creek* (Model B), and *maximum rainfall in the wettest month* (Model B). Considering that *Leptospira* can survive for weeks to months in fresh water, and are efficiently carried and disseminated by water (e.g. flooding, flowing downstream in rivers), the findings were not unexpected. Lack of metered water at home and proximity to rivers are likely to be associated with higher levels of contact with untreated freshwater, e.g. using rivers for bathing, cleaning, swimming, and recreational activities. Furthermore, poor access to water at home is generally associated with poverty (discussed below), and also influences personal hygiene, e.g. the ability to clean and wash after working outdoors, or after contact with mud, contaminated water, or animals. Two of the water-related predictors (*distance to river or major creek* and *maximum rainfall in the wettest month*) are also proxy measures of flooding risk. As seen with the post-flood leptospirosis outbreaks in 2012, flooding is an important driver of transmission in Fiji, as it is in many parts of the world.

Two of the predictors in Model B relate to livestock exposure: *total cattle density in the Tikina* and *presence of pigs in the community*. Data on cattle density in Tikinas includes both commercial and subsistence farming, and varied from < 1 to over 30 heads of cattle per square km. Infection risk could be related to direct occupational contact with cattle, or through more general contamination of the environment (especially rivers) with cattle urine. As shown in Table 5, many households in Fiji keep subsistence livestock. Backyard piggeries are commonly found in communities in Fiji and other Pacific Islands, and are usually small pens with less than 10 pigs. The pens are often built on the edge of rivers and streams to allow convenient drainage of waste, but unfortunately also lead to contamination of freshwater at that community as well as further downstream. In American Samoa, similar backyard piggeries have been associated with the risk of human leptospirosis infection [8,43]. Dairy farmers are known to be at high risk for leptospirosis in many parts of the world because of close contact with cattle, and exposure to urine during milking. In our study, high density of dairy farms was strongly associated with infection risk, but was not included as a variable in the final model because data were only available for ~70% of the Tikinas in our study. As more data on dairy farms become available, associations with leptospirosis risk could be further explored and model performance potentially improved. Commercial dairy and beef farming could potentially intensify in the future with population growth, and increase the risk of leptospirosis.

Model B also shows that leptospirosis is a disease of poverty in Fiji and disproportionately affects the poorest. Leptospirosis has been associated with poverty in diverse settings around the world, including Brazilian and Indian urban slums [33,44,45], Peruvian Amazon [39], and areas of poor socioeconomic status in the USA and Europe [46,47]. Furthermore, the combination of poverty, livestock keeping, and global climate change are important drivers of zoonotic diseases transmission [48]. In our study, participants living in communities with high poverty rates (defined as ≥40% of households in the community) had almost twice the infection rate compared to other communities, independent of the other predictors in Model B. As discussed above, poor access to metered (treated) water at home was associated with a higher risk of infection for many reasons, and is also a proxy measure of socioeconomic status.

Although serovar Pohnpei was associated for 84.2% of reactive MATs, there were differences in serovar distribution by age and by region of residence, suggesting that the relative importance of animal species in disease transmission varies between subgroups. Variation in risk factors between age groups likely relates to age-specific behaviours, e.g. young children spend more time playing around the home, and have closer contact with pets and soil; teenagers have more frequent recreational freshwater contact from swimming in rivers and waterfalls; and adults have more intense contact with livestock through occupational exposure and managing animals at home. Variation in risk factors between regions likely relates to differences in environmental settings, with proportionately greater urbanization in the Central division, and more farming in the other regions. For example, rodents could be more important in transmission cycles in urban and peri-urban areas, and livestock more important in rural areas.

Many of the risk factors and environmental drivers identified in our study provide significant cause for concern about future risk of leptospirosis in Fiji, as well as other Pacific Islands with similar environments. Population growth is typically associated with agricultural intensification, leading to increase in livestock numbers (both commercial and subsistence) and occupational exposure. With global climate change, extreme weather events and flooding are predicted to become more frequent and intense in the Pacific Islands. Rapid population growth in developing countries is often associated with urban and peri-urban slums where diseases of poverty proliferate. Although our study found that leptospirosis seroprevalence was lower in urban areas, poverty rate was a significant risk factor independent of urban or rural settings. Climate change, flooding, population growth, urbanization, and agricultural intensification may independently, or potentially synergistically, lead to enhanced leptospirosis transmission in Fiji [3].

The findings should be considered in light of the study’s limitations. Limitations of the MAT have been well documented; the test is considered to be serogroup rather than serovar specific, cross-reactions occur between serovars within a serogroup, and complex paradoxical reactions could occur in persons who have had previous infections [29]. Despite these limitations, the MAT is considered the gold standard test for identifying putative serogroups and serovars when isolates are not available [28]. Isolates of leptospires would be required to definitively confirm the serovars circulating in Fiji. Due to budgetary reasons, our study used a 6- rather than 21-serovar MAT panel to test the majority of samples. If the larger panel was used, additional less-common serovars may have been detected. However, the 6 serovars selected included the most reactive serovars when 198 randomly selected samples from this study were tested against the full 21- serovar panel; the 6 serovars selected accounted for 86.7% of the reactive samples, and one dominant serovar (Pohnpei) accounted for 65.9% of reactive samples. The reduced MAT panel size could have underestimated the overall seroprevalence by a factor of 0.13 compared to a 21-serovar panel, but unlikely to have significantly influenced the overall epidemiological patterns reported here because one serovar dominated the reactive MATs, and our data analyses in this paper were not stratified by serovars.

Our study measured antibodies to *Leptospira* to identify evidence of prior infection. However, many leptospirosis infections do not result in any apparent illness and are of no clinical significance. The severity of clinical disease depends on many factors including pathogen virulence and the individual’s immune status, comorbidities, and age [49]. Serovar Pohnpei, the serovar associated with 84.2% of MAT-reactive cases, has been reported as an important cause of overt clinical disease in the Federated States of Micronesia [32], suggesting the findings in this study are applicable not only to infection risk but also clinical illness. However, there are currently no available data on the proportion of serovar Pohnpei infections that result in clinical disease or severe complications.

Future studies could further improve our understanding of leptospirosis transmission in Fiji by examining serovar-specific risk factors; identifying the most important exposures in different subgroups such as age groups, gender, ethnic groups, and community types; determining the relative importance of livestock, rodents, pets and wildlife in transmitting leptospirosis to humans; and developing models to determine transmission (causal) pathways rather than just epidemiological links. For environmental and census variables, we used data at the place of residence, but infections could also have occurred at work or elsewhere. Future studies that focus specifically on work-related activities would provide more insight into the importance of occupational exposures in Fiji. The performances of models were partly determined by the accuracy of available environmental, census, and livestock data, and models could be updated and improved as more data become available. Models based on environmental factors, such as Model B, could be used to produce predictive risk maps for the whole of Fiji.

In summary, our study found that risk factors and drivers for human leptospirosis infection in Fiji are complex and multifactorial, and include climate, the natural environment, livestock (both subsistence and commercial), living conditions, socioeconomic status, demographics and individual behaviour. Some of these factors corroborate findings previously reported in different settings (e.g. male gender, working outdoors), but other factors appear to be specific to the cultural and environmental settings in Fiji, including ethnicity and presence of pigs in communities. By using an integrated eco-epidemiological approach and including a wide range of data sources in our analyses, we were able to quantify the relative importance of risk factors at different ecological scales. At the individual level, gender, ethnicity, and work location were strongly associated with infection risk. At the community level, important predictors of risk included rural setting, community type, poor access to clean water, close proximity to rivers, high rainfall in the wettest month, high poverty rate, presence of pigs, and high cattle density. From a wider perspective, significant geographic variations in risk and the ability to predict risk based only on environmental and census variables indicate that environmental factors play a crucial role in driving leptospirosis transmission in Fiji.

The above findings provide an important evidence base to guide the focus of public health and environmental health interventions at individual, community, and national levels. Health promotion activities and educational materials should be designed to reach the highest risk groups including males, farmers, and iTaukei. Public health and environmental health interventions should target the highest risk communities (villages, rural areas, those in hotspots and high-risk regions), and include advice on proper management of livestock, avoiding contact with floodwaters, and minimizing flooding risk (e.g. adequate garbage disposal to reduce the risk of flooding from blocked streams and drains). At high risk times, e.g. post-flooding, communities should also be reminded about the risk of leptospirosis, the use of protective measures, and the importance of seeking early medical care if unwell. In smaller communities in Fiji, where laboratory diagnostic tests are often not available, the predictive risk chart shown in Fig 8 could assist clinicians with determining the likelihood (pre-test probability) of leptospirosis infection based on a combination of individual-level variables. Broader environmental factors (both natural and anthropogenic) play a major role in leptospirosis transmission in Fiji, most of which are beyond the immediate control of individuals or small communities. Effective environmental health management at the public health and national level will therefore be crucial for the sustainable control of leptospirosis in Fiji and other countries with similar environmental and socio-demographic settings.

## Supporting Information

S1 Appendix Initial 21 pathogenic serovars included in the microscopic agglutination test (MAT) panels, and the six serovars chosen for the final MAT panel. (DOCX)

S2 Appendix Independent variables stratified by data source and scale of ecological influence. (DOCX)

S1 Checklist STROBE statement for cross-sectional studies. (DOC)
