# Supplementary material for: Human Leptospirosis Infection in Fiji: An Eco-epidemiological Approach to Identifying Risk Factors and Environmental Drivers for Transmission
Source: PLoS Negl Trop Dis. 2016 Jan 28;10(1):e0004405. doi: 10.1371/journal.pntd.0004405 (PMC4731082; doi:10.1371/journal.pntd.0004405)
Supplement: S1 Appendix — (DOCX) [file pntd.0004405.s001.docx]

S1 Appendix. Initial 21 pathogenic serovars included in the microscopic agglutination test (MAT) panels, and the six serovars chosen for the final MAT panel.

| **Species** | **Serogroup** | **Serovar** | **198 randomly selected samples from this study^#^:  % of seropositive reactions associated with each serovar** | **199 *Leptospira* ELISA-positive samples*: % of seropositive* reactions associated with each serovar** | **Used in final 6-serovar panel** |
| --- | --- | --- | --- | --- | --- |
| Interrogans | Australis | Australis | 7.3% | 1.5% | ✓ |
| Interrogans | Australis | Pohnpei | 65.9% | 51.5% | ✓ |
| Interrogans | Autumnalis | Autumnalis |  |  |  |
| Borgpetersenii | Ballum | Ballum | 3.7% | 6.1% | ✓ |
| Interrogans | Bataviae | Bataviae |  |  |  |
| Interrogans | Canicola | Canicola | 4.9% | 10.6% | ✓ |
| Weilii | Celledoni | Celledoni |  | 4.5% |  |
| Kirshneri | Cynopteri | Cynopteri | 1.2% |  |  |
| Interrogans | Djasiman | Djasiman |  |  |  |
| Interrogans | Grippotyphosa | Grippotyphosa |  |  |  |
| Interrogans | Hebdomadis | Hebdomadis |  |  |  |
| Interrogans | Icterohaemorrhagiae | Copenhageni | 4.9% | 21.2% | ✓ |
| Borgpetersenii | Javanica | Javanica |  |  |  |
| Borgpetersenii | Mini | Mini | 3.7% |  |  |
| Noguchii | Panama | Panama | 7.3% |  |  |
| Interrogans | Pomona | Pomona |  |  |  |
| Interrogans | Pyrogenes | Pyrogenes |  | 1.5% |  |
| Santarosai | Shermani | Shermani |  |  |  |
| Weilii | Sarmin | Sarmin | 1.2% |  |  |
| Interrogans | Sejroe | Hardjo |  | 3.0% | ✓ |
| Borgpetersenii | Tarassovi | Tarassovi |  |  |  |
| **TOTAL** |  |  | 100% | 100% |  |

^#^ Overall, 32.3% of samples reacted to at least one of the 21 serovars used in the MAT panel; 17.7% with MAT titre of 1:50, 9.1% 1:100; 9.1% 1:200; 5.1% 1:400; 0.5% 1:800.
**Leptospira* ELISA-positive samples from patients with suspected clinical leptospirosis from April 2012 to November 2013 (spanning both epidemic and endemic periods). Samples were systematically selected from a total of 570 samples collected during this period; selection process was designed to maximise the probability of identifying the most common serogroups from all Divisions (geographic spread) and over the entire time period (temporal spread). Overall, 33.2% of samples reacted to at least one of the 21 serovars used in the MAT panel; 4.5% with MAT titre of 1:50; 10.5% 1:100; 5.5% 1:200; 7.0% 1:400; 4.0% 1:800; 1.0% 1:1600; 0.5% 1:6400.
