# Supplementary material for: Human Leptospirosis Infection in Fiji: An Eco-epidemiological Approach to Identifying Risk Factors and Environmental Drivers for Transmission
Source: PLoS Negl Trop Dis. 2016 Jan 28;10(1):e0004405. doi: 10.1371/journal.pntd.0004405 (PMC4731082; doi:10.1371/journal.pntd.0004405)
Supplement: S2 Appendix — (DOCX) [file pntd.0004405.s002.docx]

S2 Appendix. Independent variables stratified by data source and scale of ecological influence

|  | **Data source** | |
| --- | --- | --- |
| **Scale of ecological influence** | **Questionnaires** | **Derived using geographic information systems (GIS)** |
| **Individual-level** | Age  Sex  Country of birth  Ethnicity  Religion  Highest school level completed  Occupation  Farming – none, part-time, full-time  If farmer, type(s) of animal(s)  Relative altitudes of home and farm  Availability of soap & water at lunch  Wash hands with soap & water after lunch  Availability of soap & water at dinner  Wash hands with soap & water after dinner  Type of toilet at school or work  Availability of soap & water at school or work  Wash hands with soap & water after toilet at school or work  Swimming, playing, or bathing in flood water  Walking in flood water  Contact with freshwater – recreation, walking, washing clothes, washing dishes  Sighting rats or mice at home  Physical contact with rats or mice  Sighting mongoose at home  Physical contact with mongoose  Bitten by ticks or fleas  Heard of leptospirosis before this study  Diagnosed with leptospirosis  Contacts (family, friends, colleagues) diagnosed with leptospirosis |  |
| **Household-level** | Number of household members  Household income  Source(s) of drinking water  Method(s) used to treat drinking water  Availability of tap water in house  Supply of government treated water to house  Indoor shower or tap for washing  Type of toilet at home (if any)  Location of home toilet  Sharing home toilet with other households  Availability of soap & water at home toilet  Wash hands with soap & water after using home toilet  House construction material  Floor construction material  Floor raised at least 30cm above ground  Number of rooms in house  Garbage disposal method  Stream or river near home (in community, or within 100m)  Flooding at home  Flooding of land around home  Presence of animal species at home  Grow crops, fruits, vegetables at home | Distance to rivers or major creeks  Elevation above sea level  Slope  Road density  Rainfall (multiple measures including maximum, minimum, average)  Temperature (multiple measures including maximum, minimum, average)  Land use  Soil type |
| **Community-level** | Community type  Urban rural classification  Presence of animal species in community  Grow crops, fruit, vegetables in community | Educational attainment  House construction  Sources of income (subsistence, salaried)  Ethnicity  Water supply  Electricity  Toilets  Population density  Population growth  Poverty rate  Poverty gap  Commercial beef – numbers of animals and farms  Commercial dairy – numbers of animals and farms  Subsistence beef – numbers of animals and farms  Subsistence dairy – numbers of animals and farms  Total cattle – numbers of animals and farms  Pigs – numbers of animals and farms  Goats – numbers of animals and farms  Horses – numbers of animals and farms  Sheep – numbers of animals and farms  Poultry – numbers of animals and farms  Duck – numbers of animals and farms |
